# Benefits of Mentoring in Oncology Education for Mentors and Mentees: Pre-Post Interventional Study of the British Oncology Network for Undergraduate Societies' National Oncology Mentorship Scheme

**DOI:** 10.2196/48263

**Published:** 2023-09-11

**Authors:** Taylor Fulton-Ward, Robert Bain, Emma G Khoury, Sumirat M Keshwara, Prince Josiah S Joseph, Peter Selby, Christopher P Millward

**Affiliations:** 1 Institute of Immunology and Immunotherapy College of Medical and Dental Sciences University of Birmingham Birmingham United Kingdom; 2 School of Medicine Newcastle University Newcastle upon Tyne United Kingdom; 3 Academic Cancer Sciences Unit University Hospital Southampton Southampton United Kingdom; 4 Institute of Systems, Molecular, & Integrative Biology University of Liverpool Liverpool United Kingdom; 5 University of Liverpool Liverpool United Kingdom; 6 University of Leeds Leeds United Kingdom; 7 University of Lincoln Lincoln United Kingdom

**Keywords:** mentoring, medical education, oncology, medical student, teaching, undergraduate, graduate, student, cancer, mentor, mentee, mentors, mentees

## Abstract

**Background:**

Formal education of oncology is lacking in many undergraduate medical curricula. Mentoring schemes can expose participants to specific areas of medicine and may address the shortfalls in oncology education. Few mentoring schemes have been designed within the United Kingdom, especially within oncology. There is a need to understand reasons for mentor and mentee participation in such schemes and to identify ways to minimize barriers to engagement.

**Objective:**

This study identifies motivations for participation in an oncology mentoring scheme and its benefits and limitations to both the mentee and the mentor.

**Methods:**

The British Oncology Network for Undergraduate Societies launched a National Oncology Mentorship Scheme (NOMS) on September 1, 2021. Mentees (medical student or foundation doctor) were paired with mentors (specialty registrar or consultant), for 6 months of mentoring. In total, 86 mentors and 112 mentees were recruited to the scheme. The mentees and mentors were asked to meet at least 3 times during this period and suggestions were provided on the content of mentoring. Mentees and mentors were invited to complete a prescheme questionnaire, exploring motivations for involvement in the scheme, current experiences within oncology, and knowledge and interests in the field. At the end of the scheme, mentors and mentees were asked to complete a postscheme questionnaire exploring experiences and benefits or limitations of participation. Paired analysis was performed using the Wilcoxon signed-rank test. For free text data, content analysis was applied to summarize the main themes in the data.

**Results:**

Of the 66 (59%) mentees who completed the prescheme questionnaire, 41 (62%) were clinical, 21 (32%) preclinical medical students, and the remainder were junior doctors. For mentees, networking was the primary reason for joining the scheme (n=25, 38%). Mentees ranked experience of oncology at medical school at 3 on 10 (IQR 2-5). In this, 46 (53%) mentors completed the prescheme questionnaire, 35 (76%) were registrar level, and the remainder were consultant level (n=11). The most common reason for mentor participation was to increase awareness and interest in the field (n=29, 63%). Of those who completed the prescheme questionnaire, 23 (35%) mentees and 25 (54%) mentors completed the postscheme questionnaire. Knowledge in all areas of oncology assessed significantly increased during the scheme (*P*<.001). Most mentees (n=21, 91%) and mentors (n=18, 72%) felt they had benefited from the scheme. Mentees cited gaining insights into oncology as most beneficial; and mentors, opportunities to develop professionally. Whilst mentees did not report any barriers to participating in the scheme, mentors stated lack of time as the greatest barrier to mentoring.

**Conclusions:**

British Oncology Network for Undergraduate Societies’ NOMS is expanding and is beneficial for mentees through increasing knowledge, providing exposure, and career advice in oncology. Mentors benefit from improving their mentoring skills and personal satisfaction.

## Introduction

During their careers, all doctors will be responsible for the care of patients with cancer [1,2]. Medical students and foundation doctors should be prepared for recognizing and holistically managing patients with cancer [3]. Cancer is considered a key area of practice within the incoming UK medical licensing assessment (UKMLA) [4]. There is underrepresentation of oncology within the taught curriculum and students consider oncology teaching and exposure to be lacking [5-7].

Exposure to medical specialties has been shown to be key in the formation of career intentions, and lack of teaching or exposure can act as a barrier to these specialties [8-11]. One suggested intervention to improve this exposure is facilitated, longitudinal mentoring delivered by seniors to junior or student clinicians [12-14]. Mentoring is the process of informal knowledge transmission by an experienced senior (mentor) to a more junior colleague (mentee) over a prolonged period and is often career focused [15,16]. Goals are often set depending on the mentee's preferred outcomes, and mentors use their experiences, resources, and knowledge to guide these objectives [17]. It differs from other similar learning techniques, such as coaching and sponsoring, by time span and goals [15]. Many benefits to mentoring have been reported for both the mentor and the mentee. The mentor may benefit from personal development, experience in teaching, building one’s own portfolio, and personal satisfaction [12]. Mentees may benefit from participation in research, development of professionalism, and exposure to a particular specialty or career path, among others [12].

Few mentoring programs have been designed in the United Kingdom to support medical students and junior doctors in their career development, particularly within oncology [18,19]. Existing mentoring schemes have developed questionnaires focused on determining the benefits of mentoring for mentees but not mentors [18]. Research is required to understand the motivations of mentors, why mentors participate, and how barriers preventing engagement of mentors and mentees can be removed. A description of the medium to long term impact on knowledge and interest in oncology is also needed.

The British Oncology Network for Undergraduate Societies (BONUS) implemented a National Oncology Mentorship Scheme (NOMS) in the autumn of 2021 [20]. The aim of this study is to describe the development of NOMS, to discuss applicant motivations, and to investigate the benefits and limitations to the mentor and the mentee from participating in the scheme.

## Methods

### Description of Mentoring Scheme

BONUS is a national network of medical students and junior doctors who provide educational resources and career exposure into all subspecialties of oncology. BONUS launched a NOMS on September 1, 2021, to conduct a pre-post interventional study. Mentors and mentees were recruited via social media platforms (Facebook, Meta Platforms Inc; Twitter, Twitter Inc; Instagram, Meta Platforms Inc), BONUS mailing lists, and through the mailing lists of several professional organizations and societies (Royal College of Radiologists, Association of Cancer Physicians, the National Oncology Trainees Collaborative for Healthcare Research [21], the British Association of Surgical Oncology, and the European Society of Surgical Oncology-ESSO Young Surgeons and Alumni Club). Mentees and mentors were paired based on location and, where possible, by interests. BONUS provided the mentor’s contact details to the mentee, and it was the mentee’s responsibility to contact the mentor. The mentoring itself took place over a period of 6 months and activities could be flexible depending on what best suited the mentor and mentees. The mentees and mentors were asked to meet at least 3 times during this period and suggestions were provided for the content of mentoring, for example, shadowing ward rounds or clinics, discussion of case studies or research, career advice, research proposals, etc. After the allocated period for mentoring was complete, mentees who confirmed that they met their mentor at least 3 times received a certificate of completion at the end of the scheme. Mentees were also invited to participate in an optional reflective exercise. All mentors who filled out the completion questionnaire were awarded a certificate for successfully completing the scheme.

### Participant Inclusion and Exclusion Criteria

#### Mentors

Mentors were recruited across medical, clinical, surgical, interventional, and research in oncology, and were required to be at specialty registrar (ie, receiving advanced training in their specialty after at least 4-5 years training following graduating medical school) or consultant level training (ie, after completing Certificate of Completion of Training and on the specialist register). In total 93 mentors confirmed their availability to partake in the scheme and after removing duplicates, 86 were allocated mentees. Each mentor had 1-3 mentees allocated.

#### Mentees

Mentees were either preclinical (1-2 years of undergraduate medical training), clinical (3-6 years of undergraduate medical training) medical students, or foundation doctors and were encouraged to apply for the scheme by submitting a 150-200–word statement detailing any experience they already had in oncology and why they thought they would benefit from participating in the scheme. These statements were graded according to a set criterion (Multimedia Appendix 1) and only those applicants who received a score greater than or equal to 1 were accepted onto the scheme. Members of the BONUS committee were responsible for the marking process and 2 independent individuals marked each application. Overall, there were 119 mentee applicants and 112 (94%) were recruited to the scheme.

Exclusion criteria included studying or working outside of the United Kingdom. Mentee applicants who were not studying a medical degree at the time of application were also excluded.

### Questionnaires

Prior to the commencement of mentoring, all mentors and mentees were invited to complete a noncompulsory prescheme questionnaire which detailed their motivations to participate in the scheme, their current experiences with oncology education, and mentoring, and for mentees, their knowledge and interests in oncology (Multimedia Appendix 2). Questionnaires were emailed to mentors and mentees and reminders to complete the questionnaire were sent regularly. Data were collected on Microsoft Forms. The survey questionnaires were designed through an iterative process between the project authors. No previously published or validated survey designs were used in this study. In total, 66 (59%) mentees and 46 (53%) mentors completed the prescheme questionnaire. After 6 months of allocated mentoring time was complete, mentors and mentees were asked to complete a postscheme questionnaire. Mentees were asked about their interests and knowledge of oncology, as well as their experiences and benefits or limitations from the scheme. Mentors were asked how they thought they benefited, or not benefited, from the scheme, alongside if they thought their mentee had benefited (Multimedia Appendix 2). In total, for the postscheme questionnaire, 23 paired responses were obtained from mentees and 25 paired responses from mentors (ie, completed the pre- and postscheme questionnaires).

### Analysis

Descriptive analysis was performed and results summarized as numbers and proportions for categorical data and median values with IQR for continuous data. Normality of data was assessed through a pooled approach with Shapiro-Wilk test, Jarque-Bera test, the D'Agostino K-squared test, and Anderson-Darling test. Paired analysis was performed using the Wilcoxon signed-rank test, and cases where there were missing or incomplete data were excluded from the paired analysis. *P* values of <.05 were considered statistically significant. For free text data, content analysis was applied to summarize the main themes in the data [22]. Qualitative analysis followed the process of content analysis, with the stages of data familiarization, initial coding, reviewing of codes for themes, and defining themes. An inductive approach was used throughout. The aim of this analysis was to classify and categorize the free-text data provided by participants in order to elucidate any common themes. This analysis was not designed to derive underlying meaning behind these themes. The initial coding of qualitative data was performed by RB and TFW. Excel (Microsoft Corp) was used for initial coding and sorting of the data. An inductive approach was used to generate codes. All statistical analysis was performed using R (version 4.4.0; R Core Team).

### Ethics Approval

This scheme and collection of data received ethical approval from the University of Liverpool on October 4, 2021 (reference 0154731). All participants gave informed consent in each questionnaire to having their data included. All participant identifiers were removed prior to data analysis to ensure confidentiality was maintained. Participants received no compensation for their involvement.

## Results

### Prescheme Questionnaires

#### Mentees Interests, Experiences, and Why They Joined the Scheme

In total, 66 mentees completed the prescheme questionnaire, with clinical medical students making up most mentees (n=41, 62%), followed by preclinical students (n=21, 32%) and junior doctors (n=4, 6%). Mentees rated their interest in oncology from 1 (no interest) to 5 (very interested). The median interest was 4 (IQR 4-5) and there was no significant difference between career stage and interest (*P*=.48).

For mentees, the most common reason for joining the scheme was to network with mentors (n=25, 38%), followed by gaining experience and insight into oncology as a career (n=21, 32%), and learning about oncology (n=10, 15%). A minority of mentees (n=19, 29%) described themselves as having a mentor before the scheme. Most of the mentees with preexisting mentors were clinical students (15/19, 79%).

Students were asked to rate the experience of oncology they had received throughout medical school and training on a scale of 1-10 where 1 signified no experience and 10 meaning plenty of experience. Median rating given by participants was 3 on 10 (IQR 2-5). Clinical students and junior doctors tend to rate their experience higher compared to preclinical students (4/10, IQR 3-6; 2/10, IQR 2-3, respectively, *P*<.001).

Mentees were asked which specialist areas of oncology they were most interested in learning about. The most requested areas were clinical oncology and medical oncology, with 44 (66%) mentees requesting these areas (Table 1).

**Table 1:** Count of mentees’ responses to areas of interest within oncology by their stage of training.

Mentee	Clinical oncology, n (%)	Medical oncology, n (%)	Academia and research in oncology, n (%)	Interventional oncology, n (%)	Surgical oncology, n (%)
Preclinical medical student (n=21)	10 (48)	12 (57)	14 (67)	10 (48)	15 (71)
Clinical medical student (n=41)	32 (78)	29 (71)	24 (59)	9 (22)	12 (29)
Junior doctor (n=4)	2 (50)	3 (75)	3 (75)	1 (25)	0 (0)
Total (n=66)	44 (67)	44 (67)	41 (62)	20 (30)	27 (41)

In addition, some described their areas of interest in oncology, with the top 5 most requested areas being neurological (n=22, 33%), gastrointestinal (n=18, 27%), respiratory (n=13, 20%), pediatric (n=12, 18%), and breast oncology (n=9, 14%).

#### Mentors, What They Felt They Could Offer and What They Would Gain

Of the 46 mentors, the majority were specialty registrars or equivalent (n=35, 76%), with 11 (24%) consultants. The most important reason they had for participating in the scheme was to “increase the awareness and interest in oncology” as a career (n=29, 63%), followed by 7 (15%) looking to gain “experience in medical education.” The most common activity mentors felt they could bring into their mentoring was career advice, with 45 (98%) mentors stating this. Other activities are listed in Table 2.

Mentors felt junior doctors were most likely to benefit from the scheme (n=44, 95%), followed by clinical students (n=38, 82%), and then preclinical students (n=11, 23%; Multimedia Appendix 3).

The majority of mentors (n=37, 80%) felt they would benefit from the scheme and 19 (42%) felt they would gain experience in medical education, 18 (40%) felt they would derive personal satisfaction, and 5 (11%) felt they would benefit to their portfolio.

**Table 2:** Count of what mentors felt they could contribute to the scheme by career stage.

Mentors	Clinical experience or shadowing, n (%)	Supporting with research opportunities, n (%)	Career advice, n (%)	Teaching in oncology, n (%)	Networking, n (%)	Discussion of case studies, n (%)
Consultant (n=11)	10 (91)	9 (82)	10 (91)	9 (82)	5 (45)	5 (45)
Specialty registrar (n=35)	21 (60)	24 (69)	35 (100)	32 (91)	24 (69)	29 (83)
Totals (n=46)	31 (67)	33 (72)	45 (98)	41 (89)	29 (63)	34 (74)

### Postscheme Questionnaires

#### Overview

Of those who completed the prescheme questionnaires, 23 (35%) mentees and 25 (54%) mentors completed a postscheme questionnaire. This questionnaire focused on what experience participants had in the scheme, what they gained from the scheme and what could be improved for future schemes.

#### Benefits and Limitations to Mentees Participating in the Scheme

Mentees reported how much contact they had had with their mentor over the 6-month scheme. The most common was 3-4 contacts (15/23, 65%), with 4 out of 23 (17%) receiving 1-2 contacts, 2 out of 23 (9%) receiving 5-6 contacts, and 2 out of 23 (9%) receiving 7 or more contacts. No mentees asked for less contact, 17 out of 23 (74%) stated they were happy with the number of contacts, and 6 out of 23 (26%) stated they would like more contacts. Interestingly, 21 out of 23 (91%) mentees felt they were able to build a positive rapport with their mentor, with 1 feeling unsure and 1 mentee not feeling like they had built a positive rapport.

Mentees were asked to rate their interest in oncology before and after the scheme, as well as rate their knowledge of several key roles within the oncology team. Interest in oncology among mentees was high (median 5/5 prescheme) and did not significantly change over the course of the scheme (median 4/5 postscheme, *P*=.85; Table 3). However, knowledge in all areas questioned significantly increased over the scheme (Table 3). Participants rated their knowledge of interventional oncology the lowest (both in the pre- and postscheme surveys).

Overall, 21 (91%) mentees felt they had benefited from the scheme, with 1 mentee stating “maybe” and another not describing a benefit from the scheme. When mentees who benefited from the scheme were asked to describe the most important reason which they had benefited from, four main categories emerged, which included: (1) insights into oncology as a specialty and a career (9/21, 43%); (2) direct support, networking, and mentoring from their mentor (6/21, 29%); (3) mentoring on research skills and academic (4/21, 19%); and (4) insights which confirmed oncology was not a specialty for them (2/21, 10%). For the mentees who did not benefit, this was due to difficulties connecting with their mentors.

Participants were then asked to rate their level of agreement or disagreement with 7 statements (Table 4). Responses provided were generally positive about the scheme. The statements that the mentees were in strongest agreements with were around gaining career advice, exposure to and knowledge about oncology and networking. Participants were in least agreement about the scheme increasing their participation to research.

**Table 3:** Mentees’ interest and knowledge of the different roles within oncology before and after participating in the scheme.

Variable	Prescheme questionnaire score, median (IQR)	Postscheme questionnaire score, median (IQR)	*P* value
Interest in oncology	5 (4-5)	4 (4-5)	.85
Knowledge of the members in the oncology multidisciplinary team	3 (2-4)	4 (4-5)	<.001
Knowledge of the role of medical oncologists	3 (2-4)	4 (4-5)	<.001
Knowledge of the role of clinical oncologists	3 (2-4)	4 (4-5)	<.001
Knowledge of the role of surgical oncologists	3 (2-4)	4 (4-5)	<.001
Knowledge of the role of interventional oncologists	2 (1-2)	3 (3-4)	<.001
Knowledge of the involvement of oncologists in academia or research	3 (3-4)	4 (4-5)	<.001

**Table 4:** Mentees’ responses to 7 statements surrounding benefit from the scheme.

Statement	“Strongly agree” or “agree” responses, n	Neutral responses, n	“Disagree” or “strongly disagree” responses, n
“The scheme has provided me with career advice.”	23	0	0
“The scheme has allowed me to gain an early exposure to oncology.”	20	2	1
“The scheme has widened my professional network.”	18	4	1
“The scheme has increased my knowledge of oncology.”	18	3	2
“The scheme has increased my confidence as a medical student or junior doctor.”	17	5	1
“The scheme has increased my motivation to pursue a career in oncology.”	16	5	2
“The scheme has increased my participation in research.”	10	7	6

Mentees felt the scheme would be most beneficial to them as clinical medical students (19/22), 8 of 22 as preclinical medical students, and 8 of 22 as junior doctors.

The majority of mentees (n=13) were anticipating having an ongoing relationship with their mentor, 8 were unsure, and 2 were not anticipating an ongoing relationship. In addition, 21 mentees would seek additional opportunities to work with a mentor in oncology, with 2 being unsure. Twenty-one mentees said they would recommend this scheme to a friend or a colleague, with 2 mentees stating they would “maybe” recommend this scheme.

#### Benefits and Limitations to Mentors Participating in the Scheme

In total, 18 (72%) mentors felt they had benefited from the scheme with 3 stating maybe and 4 mentors stating they had not benefited from the scheme. When asked why they felt this way, 4 main categories of reasons emerged for mentors who felt they had benefited or may have benefited. These included: (1) skills development as a mentor and as a teacher (6/21, 29%); (2) internal reflection on oncology as a career (5/21, 24%); (3) working in close proximity with engaged and committed mentees (4/21, 19%); and (4) the personal satisfaction of mentoring (3/21, 14%).

Those who did not feel they benefited felt this way due to limited engagement with their mentee (3/4), or that they already had significant mentoring roles (1/4).

The mentors were given 5 statements to rate their level of agreement or disagreement. The responses were positive, with the majority of mentors responding “strongly agree” or “agree” to the statements (Table 5).

Mentors were then asked about the barriers which may prevent them from participating in similar schemes in future. The most significant barrier was “lack of time” which was raised by 17 (68%) mentors. Other reasons included a lack of skills to be a mentor (n=3, 12%), a lack of benefit of such schemes (n=2, 8%), and lack of engagement from mentees (n=2, 8%). However, 8 (32%) mentors did not feel there were any factors which would prevent them from participating in future.

When asked if they would be a mentor in the future, 22 (88%) mentors stated they would, with 3 (12%) stating they were unsure. No mentors stated that they would not act as a mentor again in the future.

**Table 5:** Mentors’ responses to 5 statements surrounding benefit from the scheme.

Statement	“Strongly agree” or “agree” responses, n	Neutral responses, n	“Disagree” or “strongly disagree” responses, n
“I have gained personal satisfaction from participating in the scheme.”	21	4	0
“I have increased awareness and interest in oncology throughout the scheme.”	18	5	2
“I have increased my interest and experience in medical education during the scheme.”	15	9	1
“I have been able to self-reflect throughout the scheme.”	15	9	1
“I have been able to encourage research collaboration throughout the scheme.”	13	7	5

Mentors were asked how much contact they had had with their mentee during the 12-month scheme. The most common was 3-4 contacts (n=12, 48%), with 10 (40%) providing 1-2 contacts, 2 (8%) providing 5-6 contacts, and 1 (4%) providing 7 or more contacts. Notably, when asked if they would like to provide more or less contacts in the future, no mentors asked for less contacts, 17 (68%) stated they were happy with the number of contacts, and 8 (32%) stated they would like more contacts. When asked, 16 (64%) mentors felt they had built a positive rapport with their mentees, with 7 (28%) feeling unsure, and 2 out of 25 (8%) mentors stating they had not built a positive rapport.

When mentors were asked if they would participate in the scheme again or would recommend the scheme to a colleague, 20 (80%) said “yes” and 5 (20%) said “maybe.” No mentors said that they would not participate in this scheme again.

## Discussion

### Principal Findings

This study describes the impact of a national mentorship scheme within oncology on mentors and mentees. It has elucidated the reasons for participation, perceptions of oncology, and the benefits and limitations to both mentees and mentors. Benefits of participation for mentees included increased insight into all areas of oncology, provision of mentoring from their mentors and increased knowledge of research skills and academia. For mentors, key benefits were the development of skills as both a mentor and teacher, increased self-reflection, and personal satisfaction. These benefits have been shown in other mentoring programs, but never before within mentoring in oncology [23].

A key theme that emerged from this study was poor exposure to oncology throughout medical education, particularly for clinical medical students and junior doctors, consistent with previous reports [5-7]. Early exposure to specialties within medical education drives interest in that specialty and ultimately career selection and formation. Indeed, it has previously been demonstrated that increased exposure to oncology during undergraduate years results in an increased interest in a career within oncology [24]. The NOMS scheme has directly increased exposure to and knowledge around oncology for mentees. To maintain a sustainable and diverse pipeline of oncologists, scheme such as the NOMS, must be maintained [25,26].

Almost all mentees did not have formal or informal mentor prior to participating in the scheme, as found in previous research into undergraduate mentorship schemes [27]. Interestingly, the few mentees with a mentor prior to the scheme were more likely to be clinical students suggesting it is easier to access mentorship further later in medical studies. Importantly, our mentees highlighted that the ability to network with oncologists was a more compelling reason for participation in the scheme rather than to increase exposure and experience of oncology, highlighting the difficulty in obtaining a mentor as a student. Schemes such as NOMS increase accessibility to mentors and hence lead to an increased networking and interest within the specialty.

The most significant reason for mentor participation into the scheme was to increase awareness and interest of others’ into the specialty. The majority of mentors thought they could provide career advice during their sessions, alongside teaching in oncology. Mentors felt that junior doctors were most likely to benefit and preclinical students would yield the least benefit. Interestingly, most mentors believed that they would benefit from the scheme and for the most part, for intrinsic reasons (eg, gaining medical education experience and personal satisfaction). A few mentors believed they would benefit due to extrinsic reasons.

Notably, mentee knowledge across all areas of oncology increased significantly over the course of participation in NOMS, suggesting that the mentoring is an effective method of teaching in oncology. This is similar to a previous study conducted in Malaysia which demonstrated that mentoring was positively associated with talent development in a clinical setting [28]. A previous UK pilot oncology mentorship scheme also demonstrated educational benefits for mentees [18]. UK-based core medical trainees were more likely to do better on their Membership of the Royal Colleges of Physicians of the United Kingdom (MRCP) examination if they had participated in mentoring, alongside greater career progression and confidence [29].

When asked to rate their interest in oncology, students rated this high before, and after the scheme. Students applying to NOMS may be more likely to have a greater interest in oncology and mentees were expected to demonstrate an interest in oncology prior to selection. In the future, recruiting a wider range of students with lesser interest in oncology should be a priority to allow for increased uptake and interest into the specialty. Previous mentorship schemes in different specialties have shown increased interest across the course of the scheme [30,31], suggesting that the process can be effective. Despite no change in interest, the majority of mentees reported benefit from the scheme predominantly from insights into oncology as a career.

A small proportion of mentees reported that the scheme was beneficial in confirming that oncology was not a career for them (data not shown). Despite not recruiting interest into the specialty as intended, this is still a benefit to the mentee in confirming their future career prospects. Mentees believed that the scheme was useful in providing them with career advice and gaining exposure to oncology.

For those mentees who did not benefit, this was reported to be due to difficulties with contacting their mentor. Since mentors stated that the main barrier that would prevent them from participating in a similar scheme again was “lack of time,” it may be that the demands on clinical commitments make it difficult to dedicate time to mentoring. However, the majority of mentees were able to contact their mentors successfully and meet with them several times across the course of the 6-month scheme.

For mentors who benefited from participating in the scheme, they reasoned this was due to developing mentorship and teaching skills, their own personal reflection, and working alongside highly committed mentees. Similar to the mentees, those who did not benefit reported this due to limited contact or that they already had other significant mentoring roles. Therefore, limited contact throughout mentoring appears to be a barrier to its success and future programs should aim to limit this.

### Limitations

We experienced a loss of follow-up in the questionnaires, since not all mentees and mentors completed the pre- and postscheme questionnaires, despite sending regular reminders. This creates the possibility of censoring, and nonresponse bias. Additionally, there may have been a selection bias within our cohort as students had high levels of prescheme interest. The relatively small sample size of this study also presented some statistical limitations and limited the testing strategies available. Mentees were asked to demonstrate their interest in oncology prior to recruitment, and advertisement of the scheme was done using oncology-specific societies and organizations. In the future, efforts should be made to provide activities for different levels of interest in oncology to remove barriers to engagement in oncology. This study is descriptive and did not investigate the specific content areas discussed within mentoring sessions. Future work could use qualitative methodologies to investigate specific areas of content that mentors and mentees benefit from to develop NOMS further.

### Conclusions

We have demonstrated significant benefits to the mentee in participating in NOMS in increasing knowledge, providing exposure, and career advice in oncology. Mentors benefited from improving their mentoring skills and personal satisfaction. BONUS’ NOMS has become an established annual scheme and we are recruiting both mentors and mentees for future programs.

## Appendix

Multimedia Appendix 1 Criteria for grading of mentee 150-200 word applications to the scheme.

Multimedia Appendix 2 Pre- and postscheme questionnaires for mentors and mentees.

Multimedia Appendix 3 Count of mentor opinions on which stage of mentee would most likely benefit from mentoring.

## Abbreviations

BONUS: British Oncology Network for Undergraduate Societies
MRCP: Membership of the Royal Colleges of Physicians of the United Kingdom
NOMS: National Oncology Mentorship Scheme
UKMLA: UK medical licensing assessment

## Data Availability

The data sets generated and analyzed during this study are available from the corresponding author on reasonable request.
