# Supplementary material for: Benefits of Mentoring in Oncology Education for Mentors and Mentees: Pre-Post Interventional Study of the British Oncology Network for Undergraduate Societies' National Oncology Mentorship Scheme
Source: JMIR Med Educ. 2023 Sep 11;9:e48263. doi: 10.2196/48263 (PMC10520773; doi:10.2196/48263)
Supplement: Multimedia Appendix 1 [file mededu_v9i1e48263_app1.docx]

| **Criteria** | **0 points** | **1 point** | **2 points** | **3 points** |
| --- | --- | --- | --- | --- |
| Motivation and demonstration of engagement with the scheme | Demonstrates no motivation for participating in the scheme. Does not appear to have any interest in engaging with the requirements of the scheme. | Demonstrates little motivation for participating in the scheme. Does not give strong evidence that they would be fully engaged with the requirements of the scheme. | Demonstrates clear interest and motivation for participating in the scheme. Although does not state, shows that they would be engaged with the requirements of the scheme. | Demonstrates excitement and high motivation for participating in the scheme. States they are prepared to fully engage with the requirements of the scheme. |
| Reflection on previous experience in oncology | Does not mention their previous experience in oncology (whether they have any experience or not). | Only lists or bullet points previous experience in oncology, without giving any insight into the benefits or drawbacks of these experiences. | Gives previous experience in oncology, or describes lack of, and attempts to reflect on these, however with only vague detail. | Details previous experience in oncology, or describes lack of, and strongly reflects on these (e.g., explains what they learnt, or how these experiences could have been better). |
| Reasons for participating in the scheme | Does not give any reason(s) for wanting to participate in the scheme. | Gives vague reason(s) for wanting to participate in the scheme, without any detail. | Details one or a couple of clear reason(s) for wanting to participate in the scheme and states how this scheme will be beneficial. | Details several reasons for wanting to participate in the scheme, giving strong arguments on how this will benefit them and their career or personal development. |

**BONUS Mentorship Scheme: Mentee Application Marking Criteria**

Total points =

(maximum 9)
