# Supplementary material for: Benefits of Mentoring in Oncology Education for Mentors and Mentees: Pre-Post Interventional Study of the British Oncology Network for Undergraduate Societies' National Oncology Mentorship Scheme
Source: JMIR Med Educ. 2023 Sep 11;9:e48263. doi: 10.2196/48263 (PMC10520773; doi:10.2196/48263)
Supplement: Multimedia Appendix 2 [file mededu_v9i1e48263_app2.pdf]

BONUS Mentorship Scheme - Mentee Pre-Questionnaire

This pre-questionnaire forms part of the data collection for a national-level research project exploring the outcomes and benefits of mentoring in undergraduate oncology education, for both the mentor and mentee. You will be asked questions about your interests and knowledge of oncology services, as well as your motivations for participating in the scheme. We have provided you with a unique ID (see email) that will allow all data to be anonymised, whilst still allowing for a paired analysis between your pre- and post-questionnaires. All data will be stored only for only as long as needed. We ask that you provide your consent at the start of the form to allow us to use your answers in this exciting project! If you feel you do not want to contribute to this research at this time, please do not worry, it is optional! Please see the document attached to the email sent with this link for more details.

* Required

1. I confirm that I have read and have understood the information sheet dated 26/08/2021 for the above study, or it has been read to me. I have had the opportunity to consider the information, ask questions and have had these answered satisfactorily. *

   ◯ I agree

2. I understand that taking part in the study involves completing a questionnaire to evaluate the role of mentorship schemes within oncology. *

   ◯ I agree

3. I understand that my participation is voluntary and that I am free to stop taking part and can withdraw from the study at anytime without giving any reason and without my rights being affected. In addition, I understand that I am free to decline to answer any particular question or questions. *

   ◯ I agree

4. I understand that I can ask for access to the information I provide, and I can request the destruction of that information ifI wish at any time within four weeks of completing the questionnaire. I understand that following four weeks, I will no longer be able to request access to or withdrawal of the information I provide. *

◯ I agree

5. I understand that the information I provide will be held securely and in line with data protection requirements at the University of Liverpool until it is fully anonymised and then deposited in the archive for sharing and use by other authorised researchers to support other research in the future. *

◯ I agree

6. I understand that signed consent forms and questionnaires will be retained on Microsoft Forms until 1st December 2023. *

◯ I agree

7. I agree to take part in the above study. *

◯ I agree

8. Unique ID *

This will have been emailed to you alongside the link for this form. This ID will remain anonymous and will allow for paired analysis between your pre- and post-questionnaires.

Interest and knowledge of oncology

9. Are you currently a pre-clinical medical student, clinical medical student or a junior doctor? *

   ◯ Pre-clinical medical student

   ◯ Clinical medical student

   ◯ Junior doctor

10. Rate your interest in oncology before starting the mentorship scheme (5 stars = I am very interested, 1 star = I am not interested). *

    ☆ ☆ ☆ ☆ ☆

11. Rate your knowledge of: members of the multidisciplinary team in oncology services (5 stars = I know a lot, 1 star = I know very little). *

    ☆ ☆ ☆ ☆ ☆

12. Rate your knowledge of: the role of medical oncologists (5 stars = I know a lot, 1 star = I know very little). *

    ☆ ☆ ☆ ☆ ☆

13. Rate your knowledge of: the role of clinical oncologists (5 stars = I know a lot, 1 star = I know very little). *

    ☆ ☆ ☆ ☆ ☆

14. Rate your knowledge of: the role of surgical oncologists (5 stars = I know a lot, 1 star = I know very little). *

    ☆ ☆ ☆ ☆ ☆

15. Rate your knowledge of: the role of interventional oncologists (5 stars = I know a lot, 1 star = I know very little). *

☆ ☆ ☆ ☆ ☆

16. Rate your knowledge of: the involvement of oncologists in academia/research (5 stars = I know a lot, 1 star = I know very little). *

☆ ☆ ☆ ☆ ☆

17. What area(s) of oncology are you interested in learning more about? *

- [ ] Medical oncology
- [ ] Clinical oncology
- [ ] Surgical oncology
- [ ] Interventional oncology
- [ ] Academia/research in oncology

18. What fields of oncology are you interested in? (e.g., respiratory, neurological, genitourinary, gastrointestinal, etc.,) *

Motivations for participating in the mentorship scheme

19. What is the main reason you applied to this scheme? *

- ◯ To learn more about oncology
- ◯ Experience oncology to see if it suits you as a speciality
- ◯ Network with a mentor who you may be able to work with in the future (e.g., on research projects)
- ◯ See interesting patient cases and gain more clinical experience
- ◯ To build your CV
- ◯ To demonstrate your interest in this field for future job/trainee applications
- ◯ Other

20. If you selected "Other" to question 19, please specify your main reason for applying to this scheme.

21. On a scale of 1-10, how much experience in oncology do you feel you have already gained throughout your medical training? (1 = no experience, 10 = plenty of experience). *

1	2	3	4	5	6	7	8	9	10
◯	◯	◯	◯	◯	◯	◯	◯	◯	◯

22. During your medical training, what exposure to oncology have you experienced so far? *

- [ ] Pre-clinical lectures and teaching
- [ ] Oncology placement
- [ ] Other clinical placement
- [ ] Elective project
- [ ] Intercalation/Masters/PhD year(s)
- [ ] Work experience
- [ ] Other
- [ ] None

23. If you selected "Other" for question 22, please specify what exposure to oncology you have experienced during your medical training so far.

24. Do you consider yourself to have, or previously had, a formal or informal mentor in oncology? *

- ○ Yes
- ○ No

25. How many formal mentors in oncology do you consider yourself to have, or previously had? *

For example, has this mentor been allocated to you through a programme or scheme?

26. How many informal mentors in oncology do you consider yourself to have, or previously had? *

For example, is this a consultant/registrar who you consider to be a mentor but in no formal capacity?

27. Do you consider yourself to have, or previously had, a formal or informal mentor in any another field of medicine? *

- ◯ Yes
- ◯ No

28. How many formal mentors in any other field of medicine do you consider yourself to have, or previously had? *

For example, has this mentor been allocated to you through a programme or scheme?

29. How many informal mentors in any other field of medicine do you consider yourself to have, or previously had? *

For example, is this a consultant/registrar who you consider to be a mentor but in no formal capacity?

Feedback

30. Do you have any concerns before starting the mentorship scheme?

Please let us know if something could be done differently to address these either now or for next year.

31. Please leave a comment on your thoughts about the application process/form for the mentorship scheme. *

This will help us improve for future years!

32. How did you learn about the mentorship scheme? *

- ◯ Mailing list
- ◯ Facebook
- ◯ Instagram
- ◯ Twitter
- ◯ Through university society
- ◯ Through a friend

This content is neither created nor endorsed by Microsoft. The data you submit will be sent to the form owner.

Microsoft Forms

BONUS Mentorship Scheme - Mentor Pre-Questionnaire

This pre-questionnaire forms part of the data collection for a national-level research project exploring the outcomes and benefits of mentoring in undergraduate oncology education, for both the mentor and mentee. The form will ask questions about your motivations for participating and your expectations of the scheme, and will only take a couple of minutes to complete.We have provided you with a unique ID (see email) that will allow all data to be anonymised, whilst still allowing for a paired analysis between your pre-and post-questionnaires. All data will be stored only for only as long as needed. We ask that you provide your consent at the start of the form to allow us to use your answers in this exciting project! If you feel you do not want to contribute to this research at this time, please do not worry, it is optional! Please see the document attached to the email sent with this link for more details.

* Required

1. I confirm that I have read and have understood the information sheet dated 26/08/2021 for the above study, or it has been read to me. I have had the opportunity to consider the information, ask questions and have had these answered satisfactorily. *

   ◯ I agree

2. I understand that taking part in the study involves completing a questionnaire to evaluate the role of mentorship schemes within oncology. *

   ◯ I agree

3. I understand that my participation is voluntary and that I am free to stop taking part and can withdraw from the study at anytime without giving any reason and without my rights being affected. In addition, I understand that I am free to decline to answer any particular question or questions. *

   ◯ I agree

4. I understand that I can ask for access to the information I provide, and I can request the destruction of that information ifI wish at any time within four weeks of completing the questionnaire. I understand that following four weeks, I will no longer be able to request access to or withdrawal of the information I provide. *

◯ I agree

5. I understand that the information I provide will be held securely and in line with data protection requirements at the University of Liverpool until it is fully anonymised and then deposited in the archive for sharing and use by other authorised researchers to support other research in the future. *

◯ I agree

6. I understand that signed consent forms and questionnaires will be retained on Microsoft Forms until 1st December 2023. *

◯ I agree

7. I agree to take part in the above study. *

◯ I agree

8. Unique ID *

This will have been emailed to you alongside the link for this form. This ID will remain anonymous and will allow for paired analysis between your pre- and post-questionnaires.

Motivations for participating in the scheme

9. Are you a speciality registrar or equivalent, or a consultant? *

◯ Speciality registrar or equivalent

◯ Consultant

10. Have you previously participated or are you currently participating in another formal mentorship scheme? *

◯ Yes

◯ No

11. If you answered "Yes" to question 10, is this focused to oncology?

◯ Yes

◯ No

12. Have you acted, or are you acting, as a mentor in an informal capacity? *

For example, you have not been formally assigned to a mentee but provide what you consider to be a mentoring role.

◯ Yes

◯ No

13. If you answered "Yes" to question 12, is this focused to oncology?

◯ Yes

◯ No

14. At what stage(s) of medical training do you think it is most beneficial for a student/doctor to have mentorship within oncology? *

- [ ] Pre-clinical medical student
- [ ] Clinical medical student
- [ ] Junior doctor
- [ ] I do not think mentoring in oncology is beneficial

15. Which of these factors was the most important in encouraging you to participate in the scheme? *

- ○ Personal satisfaction
- ○ Research collaboration
- ○ Experience in medical education
- ○ Aim to increase quality of medical education
- ○ Opportunity for self-reflection
- ○ Aim to increase awareness and interest in oncology
- ○ Other

16. If you selected other for question 15, please specify the most important factor that encouraged you to participate in the scheme.

Expectations of the scheme

17. How many times do you expect you will be able to meet with your mentee over the course of the scheme (over a 6 month period)? *

- ◯ 1-2
- ◯ 3-4
- ◯ 5-6
- ◯ 7 or more

18. How do you expect to be able to meet with your mentee? *

- ◯ In person
- ◯ Video platform (e.g., Zoom, Microsoft Teams, etc.,)
- ◯ By phone or audio call

19. Which of these areas do you feel you may be able to bring or incorporate into your mentoring sessions? *

Select all that apply.

- ☐ Clinical experience or shadowing
- ☐ Supporting with research opportunities
- ☐ Careers advice
- ☐ Teaching in oncology
- ☐ Discussion of case studies
- ☐ Networking

20. In addition to above, what other areas do you feel you may be able to incorporate into your mentoring sessions?

Expectations of outcomes

21. Which of these areas do you expect your mentee to benefit most in from the scheme? *

- ◯ Interest and knowledge of oncology
- ◯ Aspirations to pursue a career in oncology
- ◯ Personal confidence
- ◯ Aspirations to partake in academia or research
- ◯ Widening of professional network

22. Are there any other areas you expect your mentee to benefit in?

23. Do you believe you will benefit yourself from participating in the scheme? *

- ◯ Yes
- ◯ No
- ◯ Maybe

24. If you selected "Yes" or "Maybe" for question 23, which of these areas do you expect to benefit most from by participating in the scheme?

- ◯ Research collaboration
- ◯ Experience and interest in medical education
- ◯ Personal satisfaction
- ◯ Development of own educational portfolio
- ◯ Other

25. If you selected other for question 24, please specify which area you believe you will benefit most from by participating in the scheme.

Feedback

26. How did you learn about the mentorship scheme? *

- ◯ Mailing list (e.g., BONUS, RCR, ACP, BASO, NOTCH)
- ◯ Social media
- ◯ Through a friend or colleague
- ◯ BONUS mentoring talk
- ◯ Other

27. If you selected other for question 26, please specify how you learnt about the mentorship scheme.

28. Do you have any concerns before starting the mentorship scheme? *

Please let us know if something could be done differently to address these either now or for next year.

29. Please leave a comment on your thoughts about the mentor recruitment process for the mentorship scheme. *

This will help us improve for future years!

This content is neither created nor endorsed by Microsoft. The data you submit will be sent to the form owner.

Microsoft Forms

BONUS Mentorship Scheme - Mentee Post-Questionnaire

This post-questionnaire will contribute towards the collection of data for a national research project looking at the outcomes and benefits of mentoring in undergraduate oncology education. You will be asked questions about your interests and knowledge of oncology services after having completed your mentoring, as well as your perceived benefits and feedback of the scheme. We have provided you with a unique ID (see email) that will allow all data to be anonymised, whilst still allowing for a paired analysis between your pre- and post-questionnaires. All data will be stored only for only as long as needed. We ask that you provide your consent at the start of the form to allow us to use your answers in this exciting project! If you feel you do not want to contribute to this research at this time, please do not worry, it is optional! Please see the document attached to the email sent with this link for more details.

* Required

1. I confirm that I have read and have understood the information sheet dated 26/08/2021 for the above study, or it has been read to me. I have had the opportunity to consider the information, ask questions and have had these answered satisfactorily. *

   ◯ I agree

2. I understand that taking part in the study involves completing a questionnaire to evaluate the role of mentorship schemes within oncology. *

   ◯ I agree

3. I understand that my participation is voluntary and that I am free to stop taking part and can withdraw from the study at anytime without giving any reason and without my rights being affected. In addition, I understand that I am free to decline to answer any particular question or questions. *

   ◯ I agree

4. I understand that I can ask for access to the information I provide, and I can request the destruction of that information ifI wish at any time within four weeks of completing the questionnaire. I understand that following four weeks, I will no longer be able to request access to or withdrawal of the information I provide. *

◯ I agree

5. I understand that the information I provide will be held securely and in line with data protection requirements at the University of Liverpool until it is fully anonymised and then deposited in the archive for sharing and use by other authorised researchers to support other research in the future. *

◯ I agree

6. I understand that signed consent forms and questionnaires will be retained on Microsoft Forms until 1st December 2023. *

◯ I agree

7. I agree to take part in the above study. *

◯ I agree

8. Unique ID *

This will have been emailed to you alongside the link for this form. This ID will remain anonymous and will allow for paired analysis between your pre- and post-questionnaires.

Interest and knowledge of oncology

9. Are you currently a pre-clinical medical student, clinical medical student or a junior doctor? *

◯ Pre-clinical medical student

◯ Clinical medical student

◯ Junior doctor

10. Rate your interest in oncology after completing the mentorship scheme (5 stars = I am very interested, 1 star = I am not interested). *

☆ ☆ ☆ ☆ ☆

11. Rate your knowledge of: members of the multidisciplinary team in oncology services (5 stars = I know a lot, 1 star = I know very little). *

☆ ☆ ☆ ☆ ☆

12. Rate your knowledge of: the role of medical oncologists (5 stars = I know a lot, 1 star = I know very little). *

☆ ☆ ☆ ☆ ☆

13. Rate your knowledge of: the role of clinical oncologists (5 stars = I know a lot, 1 star = I know very little). *

☆ ☆ ☆ ☆ ☆

14. Rate your knowledge of: the role of surgical oncologists (5 stars = I know a lot, 1 star = I know very little). *

☆ ☆ ☆ ☆ ☆

15. Rate your knowledge of: the role of interventional oncologists (5 stars = I know a lot, 1 star = I know very little). *

☆ ☆ ☆ ☆ ☆

16. Rate your knowledge of: the involvement of oncologists in academia/research (5 stars = I know a lot, 1 star = I know very little). *

☆ ☆ ☆ ☆ ☆

17. What area(s) of oncology are you interested in after having completed the scheme? *

- [ ] Medical oncology
- [ ] Clinical oncology
- [ ] Surgical oncology
- [ ] Interventional oncology
- [ ] Academia/research in oncology

18. What fields of oncology are you interested in after having completed the scheme? (e.g., respiratory, neurological, genitourinary, gastrointestinal, etc.,) *

Benefits of the scheme

19. Do you feel you have benefited yourself from participating in the scheme as a mentee? *

- ◯ Yes
- ◯ No
- ◯ Maybe

20. If you selected "Yes" or "Maybe" for question 19, what is the most important thing you have gained from participating in this scheme? If "No", please briefly explain why. *

21. Rate your level of agreement with the following statements *

	Strongly agree	Agree	Neither agree nor disagree	Disagree	Strongly disagree
The scheme has allowed me to gain an early exposure to oncology	○	○	○	○	○
The scheme has increased my motivation to pursue a career in oncology	○	○	○	○	○
The scheme has provided me with careers advice	○	○	○	○	○
The scheme has increased my participation in research	○	○	○	○	○
The scheme has increased my knowledge of oncology	○	○	○	○	○
The scheme has increased my confidence as a medical student/junior doctor	○	○	○	○	○
The scheme has widened my professional network	○	○	○	○	○

22. What factors, if any, may prevent you from participating in a similar scheme in the future? (e.g., time, lack of benefit, lack of interest, etc.,) *

23. Do you anticipate an ongoing relationship with your mentor? (e.g., through research projects, continued mentoring, etc.) *

- ◯ Yes
- ◯ No
- ◯ Unsure

24. Would you seek additional/other opportunities to work with a mentor in oncology in the future? *

- ◯ Yes
- ◯ No
- ◯ Maybe

25. At what stage(s) of your medical training do you feel it would be most beneficial to have a mentor in oncology? *

- ☐ Pre-clinical medical student
- ☐ Clinical medical student
- ☐ Junior doctor
- ☐ I do not feel that having a mentor in oncology is beneficial

Feedback

26. How many times did you meet with your mentor? *

- ◯ 1-2
- ◯ 3-4
- ◯ 5-6
- ◯ 7 or more times

27. Would you like to have had more, less, or a similar number of sessions with your mentor? *

- ◯ More sessions
- ◯ Fewer sessions
- ◯ Similar number of sessions

28. Do you believe you were able to build a rapport with your mentor across the six-month period of mentoring? *

- ◯ Yes
- ◯ No
- ◯ Unsure

29. What do you think worked well in the scheme? *

30. What do you think did not work well in the scheme? *

31. How can we improve the scheme for next year? *

32. Would you participate in the scheme again or recommend it to a friend? *

- ◯ Yes
- ◯ No
- ◯ Maybe

33. Any other comments?

This content is neither created nor endorsed by Microsoft. The data you submit will be sent to the form owner.

Microsoft Forms

BONUS Mentorship Scheme - Mentor Pre-Questionnaire

This pre-questionnaire forms part of the data collection for a national-level research project exploring the outcomes and benefits of mentoring in undergraduate oncology education, for both the mentor and mentee. The form will ask questions about your motivations for participating and your expectations of the scheme, and will only take a couple of minutes to complete.We have provided you with a unique ID (see email) that will allow all data to be anonymised, whilst still allowing for a paired analysis between your pre-and post-questionnaires. All data will be stored only for only as long as needed. We ask that you provide your consent at the start of the form to allow us to use your answers in this exciting project! If you feel you do not want to contribute to this research at this time, please do not worry, it is optional! Please see the document attached to the email sent with this link for more details.

* Required

1. I confirm that I have read and have understood the information sheet dated 26/08/2021 for the above study, or it has been read to me. I have had the opportunity to consider the information, ask questions and have had these answered satisfactorily. *

   ◯ I agree

2. I understand that taking part in the study involves completing a questionnaire to evaluate the role of mentorship schemes within oncology. *

   ◯ I agree

3. I understand that my participation is voluntary and that I am free to stop taking part and can withdraw from the study at anytime without giving any reason and without my rights being affected. In addition, I understand that I am free to decline to answer any particular question or questions. *

   ◯ I agree

4. I understand that I can ask for access to the information I provide, and I can request the destruction of that information ifI wish at any time within four weeks of completing the questionnaire. I understand that following four weeks, I will no longer be able to request access to or withdrawal of the information I provide. *

◯ I agree

5. I understand that the information I provide will be held securely and in line with data protection requirements at the University of Liverpool until it is fully anonymised and then deposited in the archive for sharing and use by other authorised researchers to support other research in the future. *

◯ I agree

6. I understand that signed consent forms and questionnaires will be retained on Microsoft Forms until 1st December 2023. *

◯ I agree

7. I agree to take part in the above study. *

◯ I agree

8. Unique ID *

This will have been emailed to you alongside the link for this form. This ID will remain anonymous and will allow for paired analysis between your pre- and post-questionnaires.

Motivations for participating in the scheme

9. Are you a speciality registrar or equivalent, or a consultant? *

- ◯ Speciality registrar or equivalent
- ◯ Consultant

10. Have you previously participated or are you currently participating in another formal mentorship scheme? *

- ◯ Yes
- ◯ No

11. If you answered "Yes" to question 10, is this focused to oncology?

- ◯ Yes
- ◯ No

12. Have you acted, or are you acting, as a mentor in an informal capacity? *

For example, you have not been formally assigned to a mentee but provide what you consider to be a mentoring role.

- ◯ Yes
- ◯ No

13. If you answered "Yes" to question 12, is this focused to oncology?

- ◯ Yes
- ◯ No

14. At what stage(s) of medical training do you think it is most beneficial for a student/doctor to have mentorship within oncology? *

- [ ] Pre-clinical medical student
- [ ] Clinical medical student
- [ ] Junior doctor
- [ ] I do not think mentoring in oncology is beneficial

15. Which of these factors was the most important in encouraging you to participate in the scheme? *

- ○ Personal satisfaction
- ○ Research collaboration
- ○ Experience in medical education
- ○ Aim to increase quality of medical education
- ○ Opportunity for self-reflection
- ○ Aim to increase awareness and interest in oncology
- ○ Other

16. If you selected other for question 15, please specify the most important factor that encouraged you to participate in the scheme.

Expectations of the scheme

17. How many times do you expect you will be able to meet with your mentee over the course of the scheme (over a 6 month period)? *

- ◯ 1-2
- ◯ 3-4
- ◯ 5-6
- ◯ 7 or more

18. How do you expect to be able to meet with your mentee? *

- ◯ In person
- ◯ Video platform (e.g., Zoom, Microsoft Teams, etc.,)
- ◯ By phone or audio call

19. Which of these areas do you feel you may be able to bring or incorporate into your mentoring sessions? *

Select all that apply.

- ☐ Clinical experience or shadowing
- ☐ Supporting with research opportunities
- ☐ Careers advice
- ☐ Teaching in oncology
- ☐ Discussion of case studies
- ☐ Networking

20. In addition to above, what other areas do you feel you may be able to incorporate into your mentoring sessions?

Expectations of outcomes

21. Which of these areas do you expect your mentee to benefit most in from the scheme? *

- ◯ Interest and knowledge of oncology
- ◯ Aspirations to pursue a career in oncology
- ◯ Personal confidence
- ◯ Aspirations to partake in academia or research
- ◯ Widening of professional network

22. Are there any other areas you expect your mentee to benefit in?

23. Do you believe you will benefit yourself from participating in the scheme? *

- ◯ Yes
- ◯ No
- ◯ Maybe

24. If you selected "Yes" or "Maybe" for question 23, which of these areas do you expect to benefit most from by participating in the scheme?

- ◯ Research collaboration
- ◯ Experience and interest in medical education
- ◯ Personal satisfaction
- ◯ Development of own educational portfolio
- ◯ Other

25. If you selected other for question 24, please specify which area you believe you will benefit most from by participating in the scheme.

Feedback

26. How did you learn about the mentorship scheme? *

- ◯ Mailing list (e.g., BONUS, RCR, ACP, BASO, NOTCH)
- ◯ Social media
- ◯ Through a friend or colleague
- ◯ BONUS mentoring talk
- ◯ Other

27. If you selected other for question 26, please specify how you learnt about the mentorship scheme.

28. Do you have any concerns before starting the mentorship scheme? *

Please let us know if something could be done differently to address these either now or for next year.

29. Please leave a comment on your thoughts about the mentor recruitment process for the mentorship scheme. *

This will help us improve for future years!

This content is neither created nor endorsed by Microsoft. The data you submit will be sent to the form owner.

Microsoft Forms
