# Supplementary material for: Benefits of Mentoring in Oncology Education for Mentors and Mentees: Pre-Post Interventional Study of the British Oncology Network for Undergraduate Societies' National Oncology Mentorship Scheme
Source: JMIR Med Educ. 2023 Sep 11;9:e48263. doi: 10.2196/48263 (PMC10520773; doi:10.2196/48263)
Supplement: Multimedia Appendix 3 [file mededu_v9i1e48263_app3.docx]

|  | **Pre-Clinical** (n=21) | **Clinical** (n=41) | **Junior Doctor** (n=4) |
| --- | --- | --- | --- |
| **Speciality Registrar (or equivalent)**  (n=35) | **10** | **30** | **33** |
| **Consultant**  (n=11) | **1** | **8** | **11** |
| **Totals**  (n=46) | **11** | **38** | **44** |

**Count of mentor opinions on which stage of mentee would most likely benefit from mentoring**
